# The Evolution of Pharmacist Administered Vaccinations in Australia: A Narrative Review of Legislation and Regulatory Documents

**DOI:** 10.3390/pharmacy14040092

**Published:** 2026-06-26

**Authors:** Shambel Nigussie Amare, Kwang Choon Yee, Myra Leung, Mark Naunton, Abbey Wilson, Annika Rooney, Omar Gannash, Mary Bushell

**Affiliations:** 1 Discipline of Pharmacy, University of Canberra, Canberra, ACT 2617, Australia; kwang_choon.yee@canberra.edu.au (K.C.Y.); mark.naunton@canberra.edu.au (M.N.); abbey.wilson2@health.nsw.gov.au (A.W.); annika.rooney@gmail.com (A.R.); omar.gannash100@gmail.com (O.G.); mary.bushell@canberra.edu.au (M.B.); 2 Discipline of Optometry, Faculty of Health, University of Canberra, Canberra, ACT 2617, Australia; myra.leung@canberra.edu.au

**Keywords:** vaccines, immunisation, legislation, regulations, pharmacists, Australia

## Abstract

**Background**: Since 2014, all Australian jurisdictions have progressively amended legislation to authorise pharmacists to administer vaccines, evolving from restricted pilots to an essential public health pillar. **Objective**: This review analyses the longitudinal evolution of pharmacist-administered vaccinations (PAVs), documenting changes in authorised vaccines, age eligibility, and regulatory frameworks across all Australian jurisdictions. **Methods**: A retrospective review of Australian jurisdictional legislation, regulations, and policy documents was undertaken. Searches included official legislative registers, Government Gazettes, Health Department protocols, and professional guidance published by Pharmaceutical Society of Australia (PSA) and The Pharmacy Guild of Australia between 2014 to 2026. Documents were independently reviewed by five authors, followed by secondary verification and consensus-based adjudication to resolve discrepancies and confirm findings. **Results**: PAVs scope was expanded from a single influenza pilot in 2014 to include over 21 vaccine-preventable diseases by 2026. The COVID-19 pandemic catalysed rapid reform, leading to the standardisation of age eligibility (largely ≥5 years). A landmark milestone occurred in 2025 when South Australia enabled pharmacists to administer any vaccine within their professional scope. **Conclusion**: Legislative reforms have significantly enhanced vaccine accessibility. However, jurisdictional fragmentation persists. National harmonisation, using a competency-based model similar to South Australia, is recommended to streamline delivery and optimise public health outcomes.

## 1. Background

Vaccinations are a vital component of public health, helping to avert between 3.5 and 5 million fatalities annually among people of all ages, based on estimates from the World Health Organisation (WHO) [1]. In an effort to make vaccines more accessible, several countries have expanded the scope of pharmacists’ practice, building upon their traditional responsibilities as immunisation educators and facilitators of vaccine administration in diverse healthcare settings [2]. According to a 2020 global survey, pharmacy-based vaccination is available in at least 36 countries and territories, with 26 of these countries authorising pharmacists to administer vaccines directly [3].

Australia is among the countries that permit pharmacist-administered vaccinations (PAVs). Comprising eight states and territories, each jurisdiction has its own legislation. In May 2010, the Pharmacy Guild of Australia released the Community Pharmacy Roadmap to expand the scope of pharmacists’ practice, including vaccinations [4]. Following this, on 5 December 2013, the Pharmacy Board of Australia declared that vaccination was within the scope of practice for pharmacists.

The Queensland Pharmacist Immunisation Pilot (QPIP), launched in 2014, enabled Australian pharmacists to vaccinate for the first time [5]. Over two phases of the pilot, a total of 35,558 doses of vaccines were administered. The findings of the pilot were consistent with international evaluations of PAVs, demonstrating strong community acceptance, with 96% (*n* = 10,454) of participants expressing satisfaction with the service [6]. This pilot, along with the evidence it generated, served as a catalyst for subsequent legislative amendments and the broader rollout of pharmacist vaccination services across Australia.

The introduction of legislative amendments that enabled pharmacists to administer vaccines occurred at different times across Australian jurisdictions. Western Australia (WA) was the first state to amend its legislation in December 2014 to authorise pharmacists to administer influenza vaccines to individuals aged 18 years or older [7]. In 2015, New South Wales (NSW), South Australia (SA), and the Northern Territory (NT) also implemented legislative changes [8,9,10]. In NSW, pharmacists were permitted to provide influenza immunisation to people aged 18 years of age and older [8]. In SA, the age limit was lower, allowing pharmacists to vaccinate individuals aged 16 years and older [9]. In the NT, under the Pharmacist-Led Immunisation Pilot programme (NT-PLIP), pharmacists were authorised to administer influenza, measles/mumps/rubella (MMR), and diphtheria/tetanus/pertussis (dTpa) to individuals aged 16 years and older [10,11].

By 2016, similar legislative changes were introduced in the Australian Capital Territory (ACT), Queensland (QLD), Tasmania (TAS), and Victoria (VIC) [12,13,14,15]. In the ACT [12] and TAS [13], pharmacists were authorised to administer influenza vaccines to individuals aged 18 years and older. In QLD, legislation further expanded the scope of practice, enabling pharmacists to administer influenza, measles, and pertussis vaccines to the same age group [14]. Similarly, legislation was amended in VIC, allowing pharmacists to administer influenza and pertussis vaccines to individuals aged 18 years and older.

As indicated above, different Australian states and territories amended their legislation to allow pharmacists to administer vaccines at different times. However, the vaccines authorised for administration by pharmacists are not consistent across all Australian states and territories. This variation creates confusion for consumers and healthcare professionals. Therefore, this study aims to compile a complete list of all vaccines authorised for administration by pharmacists in each Australian state and territory, along with the respective years of authorisation and the opportunities for pharmacists to administer these vaccines.

Developing a comprehensive record of pharmacist-administered vaccinations across Australia will support policymakers in each state and territory when considering future legislative amendments. Additionally, it will enhance awareness among consumers and healthcare professionals, ensuring greater clarity and accessibility of vaccination services.

## 2. Methods

### 2.1. Document Identification and Search Strategy

The study employed a retrospective longitudinal review of legislative and regulatory documents to track the evolution of PAVs across Australia. Data extraction was conducted from 15 November 2025 to 10 April 2026. Primary data were sourced from a multistage search of official government repositories and professional pharmacy bodies across all eight jurisdictions.

**Figure 1:**
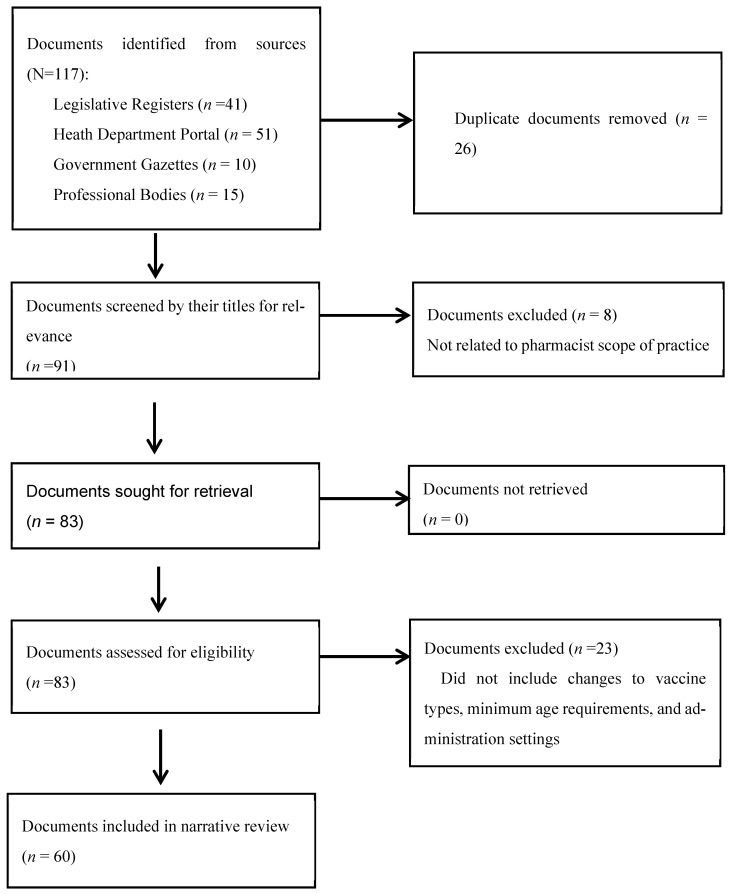
Flow diagram illustrating the number of documents identified, screened, excluded and included in the narrative review.

To identify changes in Poisons and Therapeutic Goods Acts and relevant regulations, the following specific repositories were systematically searched (Figure 1):

1. Legislative Registers: WA legislation, NSW Legislation, NT Legislation, ACT legislation registers, and Queensland Legislation
2. Heath Department Portals: SA Health, NT Health, and the Department of Health for Tasmania, Victoria, and Queensland
3. Official Gazettes: Searches of Government Gazettes (including the Victorian Government Gazette) were conducted to identify secondary legislative instruments.
4. Professional Bodies: Professional guidance documents from the Pharmaceutical Society of Australia (PSA) and The Pharmacy Guild of Australia were reviewed to contextualise legislative changes and implementation timelines.

The search was executed using standardised keywords, including pharmacist, vaccine, immunisation, legislation, regulatory scope, and poisons regulations. In instances where details were unavailable on these websites, relevant jurisdictional authorities were contacted directly to obtain further information.

### 2.2. Inclusion and Exclusion Criteria

Documents were assessed against predefined inclusion and exclusion criteria (Table 1).

## 3. Data Extraction and Analysis

A standardised data extraction template was used to systematically capture four key variables: the jurisdiction, the year of regulatory change, the specific vaccines added to the pharmacist formulary, and the minimum age of the recipient (see Supplementary Material). To ensure objectivity, five authors (SNA, MB, AW, AR, OG) independently reviewed all documents to identify regulatory shifts over the past decade. Subsequently, the remaining authors verified the identified changes, and a final consensus was reached among all authors regarding the information presented in the documents.

Historical and superseded versions of each regulation were reviewed to verify the exact commencement dates of specific policy shifts. The resulting data were then categorised and analysed to identify the broader trends, such as the progressive harmonisation of age requirements across Australian jurisdictions.

## 4. Results

### 4.1. Expansion of Pharmacist-Administered Vaccines by Type and Year

Since 2014, several key enablers have supported the expansion of PAVs across Australia. One of the most significant developments has been the progressive increase in the number and types of vaccines authorised for pharmacist administration (see Supplementary Material) [9,13,16,17,18,19,20,21,22,23,24,25,26,27,28,29,30,31,32,33,34,35,36,37,38,39,40,41,42,43,44,45,46,47,48]. In 2017, SA amended its legislation to include MMR and diphtheria–tetanus–acellular pertussis–inactivated poliovirus (dTpa/IPV) vaccines [9]. By 2018, VIC permitted pharmacists to administer MMR vaccines to individuals aged 16 years and older [16], followed by the inclusion of MMR and dTpa vaccines in NSW [39] and TAS in 2019 [17]. WA expanded its list to include MMR, dTpa, and meningococcal ACWY (MenACYW) for individuals aged 16 years and older [18].

The COVID-19 pandemic catalysed rapid expansions. In 2020, QLD authorised pharmacists to administer cholera, polio, haemophilus influenzae type B (Hib), hepatitis A (Hep A), and MenACYW, vaccines for individuals aged 16 years and older [19]. That same year, VIC approved MenACYW vaccination for individuals aged 15 years and older [20], followed by pneumococcal vaccine inclusion in QLD in 2021 [40].

In 2022, further expansion occurred. NSW permitted pharmacists to administer Hep A, hepatitis B (Hep B), human papillomavirus (HPV), MenACYW, polio, typhoid, zoster, and Japanese encephalitis (JE) vaccines [41]. WA added HPV for individuals aged 11 years and older [21], while VIC authorised HPV, JE, monkeypox, pneumococcal, and zoster vaccines [42].

In 2023, the ACT added eleven new vaccines to its pharmacist formulary [24], while the NT approved Hib, Hep A, Hep B, zoster, HPV, MenACYW, meningococcal B (MenB)**,** pneumococcal, polio, and varicella vaccines [26]. QLD expanded to include Hep B, varicella, MenB, HPV, typhoid, zoster, and JE vaccines [27], and NSW added Hib, MenB, meningococcal C (MenC), varicella, and rabies vaccines [38]. SA added JE, dTpa, Hib, polio, Hep A, Hep B, HPV, and varicella, and zoster vaccines [9], while TAS expanded its formulary to include Hib, Hep A, Hep B, HPV, JE, MenACYW, MenB, MenC, pneumococcal, polio, rabies, typhoid, varicella, and zoster vaccines [28].

In 2024, NSW authorised pharmacists to administer monkeypox, pneumococcal, rabies and respiratory syncytial virus (RSV) vaccines [29,30]. RSV vaccination was also authorised in the ACT, TAS, QLD, and VIC [13,31,32,34]. WA added Hep B, pneumococcal, polio, RSV, varicella, and zoster vaccines to the pharmacist formulary [35].

In February 2025, SA authorised pharmacists to administer any vaccine listed in the Australian Immunisation Handbook or approved by the Minister of Health, provided they operate within their individual scope of practice and are trained under the Controlled Substances (Poisons) Regulations 2011, sub regulation 18(3a) [36]. In the same year, NT authorised rotavirus and RSV vaccines for individuals aged five years and older [37].

In 2026, NSW and TAS authorised pharmacists to administer the cholera vaccine. Additionally [43,44], WA expanded its list to include MenB and MenC vaccines in the pharmacist formulary (Table 2) [49].

### 4.2. Expansion of Eligible Age Groups

Following confirmation of the safety and effectiveness of pharmacist-administered vaccines, jurisdictions progressively reduced minimum age requirements. In 2019, NSW and QLD reduced the age threshold for influenza vaccination to 16 years [39,43], while TAS authorised influenza vaccines for individuals aged 10 years and over [17]. By 2020, the ACT, SA, VIC, QLD, and NT, lowered the minimum age for influenza vaccination to 10 years [9,19,20,44,45]. VIC also reduced the age for administering dTpa and MMR vaccines to 15 years [20].

In 2022, WA authorised influenza vaccination for children aged 5 years and over, and dTpa for those aged 11 and over [21]. SA further reduced the age for influenza from 10 years to 5 years [9], while NSW authorised dTpa and MMR vaccines for those aged 12 and above, and influenza for those aged 5 and over [41].

By 2023, the ACT lowered the eligibility age for dTpa, MMR, influenza, and MenACYW vaccines to 5 years [25]. The NT reduced the minimum age for COVID-19, dTpa, influenza, and MMR vaccines to 5 years [26], and QLD lowered the age to 2 years for all PAVs except COVID-19 and influenza [33]. SA expanded the range of vaccines available for children aged 5 and over [9], while TAS authorised COVID-19, dTpa, and MMR vaccines for children aged 10 and older [28]. NSW also approved HPV vaccines for children as young as 9 years old [38]. In 2024, both WA and VIC reduced the lowest eligible age for vaccines delivered by pharmacists to five years [32,35]. In 2026, the ACT, NSW, TAS, and WA decreased the age for pharmacist-administered influenza vaccines to 2 years and above (Table 3) [46,47,48,49].

### 4.3. Integration of Pharmacists as COVID-19 Immunisation Providers

**Figure 2:**
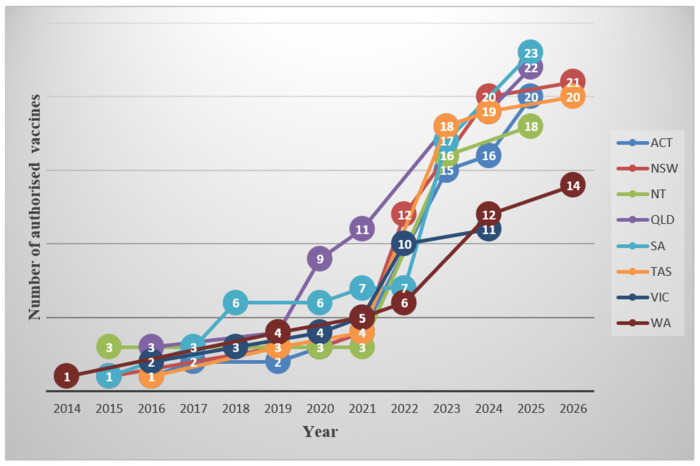
The total number of authorised vaccines for PAVs by jurisdictions (2014–2026).

Although pharmacists were initially excluded from the early stages of the COVID-19 vaccine rollout [50], all jurisdictions amended legislation in 2021 to authorise pharmacists to administer COVID-19 vaccines [9,40,51,52,53,54,55] (Table 2). The pandemic served as a critical driver of pharmacist workforce mobilisation and broader PAVs policy reform. From 21 February 2021to 31 December 2023, approximately 69.31 million doses of the COVID-19 vaccines were administered across Australia. Pharmacists administered more than 11 million doses, highlighting their central role in the national vaccination effort [56]. Prior to the pandemic, pharmacists’ vaccination activities were largely limited to influenza, dTpa, and MMR. Since then, their scope has expanded to cover over 21 vaccine-preventable diseases (Figure 2).

### 4.4. Broadening of PAVs Settings

While PAVs services initially occurred within community pharmacies, recent reforms have enabled pharmacists to deliver vaccines in a wider range of settings. By 2020, pharmacists in NSW, NT, and WA, were authorised to vaccinate within aged care homes, Aboriginal healthcare organisations, government-run and privately operated hospitals, as well as local health clinics [57,58,59]. In the same year, VIC authorised pharmacists to administer in general practices, Aboriginal Community Controlled Health Organisations, local council clinics, public and private hospitals, community health centres, staff occupational health clinics, aged care facilities, and home-based care settings [20].

In 2023, Queensland permitted pharmacists to administer vaccines in private hospitals, public sector health service facilities, medical clinics, Indigenous health organisations, and residential care homes for older adults [27]. In 2024, Tasmania expanded pharmacist vaccination services to community health services and residential aged care facilities [13].

### 4.5. Inclusion of Government-Funded Vaccines

The inclusion of state or territory-funded vaccines in pharmacists’ scope of practice has been a significant system enabler for expanding PAVs in Australia. Starting in 2019, Tasmania added state-funded MMR vaccines for individuals aged 16 and over [17], and a trial in the ACT authorised pharmacists to administer National Immunisation Program (NIP) influenza vaccines to those aged 65 and over, paving the way for broader adoption [60]. By 2020, pharmacists in the ACT were officially allowed to administer NIP-funded vaccines [61], with similar strategies extending to NSW, Tasmania, Victoria, WA, and SA [9,60,62]. Free influenza vaccination campaigns in WA [63] and Queensland [64,65] further encouraged pharmacist contributions.

The introduction of the National Immunisation Program Vaccinations in Pharmacy (NIPVIP) initiative on 1 January 2024 in community pharmacy settings represents another key enabler for the expansion of PAVs. The programme is designed to enhance vaccine accessibility for patients and reduce the financial burden associated with immunisation, ultimately helping to protect Australians from vaccine-preventable diseases [66]. As of 29 April 2024, the programme was further expanded to include the administration of free National Immunisation Program (NIP) vaccines in residential aged care and disability homes. Eligibility for receiving NIP vaccines under this programme requires individuals to be aged 5 years and over, in accordance with relevant state or territory legislation.

## 5. Discussion

This study provides a comprehensive overview of the evolving scope of PAVs services in Australia, highlighting significant legislative developments across all states and territories from 2014 to early 2026. The findings illustrate that pharmacists have increasingly become key immunisation providers, supported by systematic expansion in vaccine authorisation, eligible age groups, practice settings, and access to government-funded vaccines. This evolution mirrors a global shift toward task-sharing in primary care, aligning Australia with international models such as the United States and Canada, where the pharmacy workforce has been successfully leveraged to meet national public health goals [67,68].

One of the most prominent trends observed is the progressive expansion of the pharmacist formulary. Initially limited to a small number of vaccines such as influenza, MMR, and dTpa, the list of vaccines that pharmacists are authorised to administer has grown substantially since 2017. Notably, the integration of pharmacists into the COVID-19 vaccine rollout in 2021 served as a catalyst for broader legislative reform. All jurisdictions amended regulations to authorise pharmacist-led COVID-19 vaccinations, resulting in over 11 million doses being administered by pharmacists by the end of 2023 [56]. This marked a turning point in recognition of pharmacists” role in immunisation service delivery.

Concurrently, there has been a national trend toward lowering the minimum age for PAVs. Whereas earlier authorisations restricted services to individuals aged 16 years and above, many jurisdictions now allow vaccinations for children as young as 5, and in Queensland, even as young as 2 from 2025 [33]. This reduction reflects growing confidence in pharmacists’ capability to safely provide vaccines to younger populations, supported by appropriate training, standards, and regulatory oversight [36]. By demonstrating that task-sharing can be safely applied to paediatric populations, Australia provides a blueprint for other nations looking to expand vaccination coverage through pharmacy networks.

This review also highlights the broadening of PAVs service settings beyond traditional community pharmacies. In several states, pharmacists are now authorised to vaccinate in aged care homes, Indigenous healthcare organisations, both private and public hospitals, and local community-based clinics [57,58,59]. These developments address fundamental flaws in traditional healthcare delivery—specifically limited accessibility for vulnerable and underserved populations in rural or remote areas. By decentralising vaccine delivery, jurisdictions have effectively created a high-density immunisation net that reaches beyond the clinical clinic model.

Access to publicly funded vaccines has been another critical enabler of PAVs expansion. The progressive inclusion of pharmacists in the NIP has helped reduce cost barriers and legitimised pharmacists’ role within the public health system [9,60,62]. The implementation of the NIPVIP initiative in 2024 institutionalised this role by providing free NIP vaccines in both community pharmacies and off-site settings such as disability homes [66]. Compared to international models where funding is often a primary barrier to PAVs uptake, the Australian NIP integration represents a major policy milestone that anchors the profession within the broader national health strategy [69].

Despite these achievements, the expansion of PAVs services has not occurred uniformly across jurisdictions. Fragmented implementation timelines between states and territories have continued to create variability in pharmacists’ scope of practice. For example, while SA now permits pharmacists to administer any vaccine listed in the Australian Immunisation Handbook, other jurisdictions except QLD continued to rely on discrete formulary updates and age-specific restrictions) [8,9,10,12,13,15,16,17,18,19,20,21,22,23,24,25,26,27,28,29,30,31,32,33,34,35,36,37,38,39,40,41,42,43,45,46,48,49,51,53,54,55,70,71,72,73,74,75,76,77,78,79,80].

The reasons underlying this variation are likely multifactorial. Unlike nationally coordinated immunisation programmes, regulation of pharmacist vaccination services remains largely the responsibility of individual states and territories [81], resulting in differing approaches to scope-of-practice expansion. Variation in regulatory frameworks may reflect differences in local healthcare priorities, workforce availability, stakeholder engagement, and jurisdictional attitudes towards professional role expansion. While such flexibility allows jurisdictions to respond to local circumstances, it has also contributed to uneven implementation of pharmacist vaccination services across Australia. The persistence of regulatory fragmentations raises important policy considerations [82]. Difference in vaccine authorisations, age eligibility criteria, and practice settings may create administrative complexity for pharmacists, particularly those practising across multiple jurisdictions, and may contribute to public uncertainty regarding vaccine availability. Furthermore, unequal access to pharmacist-administered vaccines between jurisdictions may result in disparities in service availability and vaccination opportunities. These findings suggest that greater national alignment of vaccination regulations could improve consistency, facilitate workforce mobility and strengthen the contribution of pharmacists to national immunisation objectives.

Beyond legislative reforms, the expansion of PAVs services has important implications for healthcare delivery and public health outcomes [83]. Community pharmacies are among the most accessible healthcare settings and often provide extended opening hours without the need for appointments [81]. Consequently, pharmacist vaccination services have the potential to reduce access barriers [83,84], particularly for individuals in rural and remote communities, those with limited access to primary care, and populations that may be underserved by traditional healthcare models.

The rapid integration of pharmacists into the COVID-19 vaccination programme further demonstrated the capacity of the profession to support large-scale immunisation efforts. The administration of more than 11 million COVID-19 vaccine doses by pharmacists by the end of 2023 highlights the contribution of community pharmacists to vaccination delivery [56]. This experience reinforces the potential value of pharmacists in enhancing vaccination coverage, increasing system capacity, and improving responsiveness during public health emergencies. Moreover, previous studies have reported high levels of patient acceptances and satisfaction with PAVs services [6,85], suggesting that expanded pharmacist involvement may contribute not only improved access but also to positive patient experiences and engagement with preventive healthcare services. 

### Limitations of This Review

The data in this review show the dynamic and evolving nature of legislation and regulations across Australian jurisdictions. However, it does not provide professional practice advice; therefore, pharmacists must ensure that their practice adheres to the updated recommendations in the Australian Immunisation Handbook and professional practice guidelines for service delivery [86], as well as to their jurisdiction’s regulations and standards.

This review relied primarily on publicly available legislative and regulatory documents. While every effort was made to identify and verify the most current information, there is a possibility that some documents may not fully reflect recent amendments, unpublished implementation guidance, or jurisdiction-specific operational arrangements. Consequently, minor inaccuracies or omissions may exist due to differences in document availability, interpretation, and timing of publications.

In addition, legislative or regulatory approval does not necessarily translate into immediate implementation in practice. Delays may occur between regulatory authorisation and service delivery because of workforce training requirements, developments of practice protocols, funding arrangements, supply chain considerations, and organisational readiness. Therefore, the practical availability of pharmacist-administered vaccination services may not always align with the timing of legislative changes reported in this review.

Finally, the regulatory landscape for pharmacist-administered vaccination in Australia continues to evolve rapidly. As jurisdictions regularly update authorisations, age eligibility criteria, funding arrangements, and service delivery settings, some findings presented in this review may have a relatively short shelf-life and require ongoing monitoring and periodic updates to maintain currency.

## 6. Conclusions

Since the introduction of pharmacist-administered vaccination in 2014, the scope of these services in Australia has expanded significantly. Initially limited to influenza vaccines, pharmacists have gained the authority to administer a wide range of vaccines. This evolution has included reduced age restrictions, an increase in the variety of vaccines pharmacists can administer, broader settings for PAVs, and improved access to NIPVIP-funded vaccines. These advancements have strengthened pharmacists’ role in immunisation, enhancing accessibility and public health outcomes across the country.

Despite this progress, substantial variation remains between jurisdictions regarding vaccine authorisations, age eligibility requirements, and service delivery arrangements. Future research should examine opportunities for greater harmonisation of vaccination regulations across Australia to support consistency in service provision and reduce regulatory complexity. Additional research is also needed to explore workforce development requirements, including training, credentialing, and support mechanisms that enable pharmacists to safely expand their immunisation roles. Furthermore, evaluations of vaccine uptake and cost-effectiveness are required to better understand the long-term public health impact of pharmacist-administered vaccination services and inform future policy development.

## Figures and Tables

**Table 1:** Inclusion and exclusion criteria.

Criteria	Inclusion	Exclusion
Document type	Acts of Parliament, Regulations, Protocols, Gazettes, Official Health Guidelines	Internal pharmacy business policies
Geography	All Australian states and territories	International pharmacy regulations or non-Australian jurisdictions
Timeline	Documents published or in effect between December 2014 to April 2026	Documents issued before December 2014 that were not in effect during the study period
Content	Changes to vaccine types, minimum age requirements, administration settings, and funding eligibility	General pharmacy business regulations unrelated to clinical vaccination services

**Table 2:** Expansion of pharmacist-administered vaccines across Australian jurisdictions.

Year	State/Territory	Vaccine Added for PAVs
2017	SA	MMR, dTpa/IPV
	ACT	dTpa
2018	SA	MenB, MenC
	VIC	MMR
2019	NSW, TAS	MMR, dTpa
	QLD	dTpa/IPV
	WA	MMR, dTpa, MenACYW
2020	QLD	Cholera, polio, Hib, Hep A, MenACYW
	VIC	MenACYW
	ACT	MMR
2021	ACT, NSW, NT, SA, TAS, WA	COVID-19
	QLD	COVID-19, pneumococcal
2022	NSW	Hep A, Hep B, HPV, MenACYW, polio, typhoid, zoster, JE
	WA	HPV
	VIC	HPV, JE, Monkeypox, pneumococcal, zoster
2023	ACT	Hep A, Hep B, HPV, MenACYW, Polio, Typhoid, Zoster, Hib, MenB, MenC, varicella
	NSW	Hib, MenB, varicella, MenC
	NT	Hib, Hep A, Hep B, HPV, JE, Monkeybox, MenB, MenC, MenACYW, Pneumococcal, Polio, Rabies, Varicella, Zoster
	QLD	Hep B, HPV, JE, MenB, typhoid, varicella, zoster
	SA	dTpa, Hib, HepA, HepB, HPV, JE, polio, varicella, zoster, pneumococcal
	Tas	Hib, Hep A, Hep B, HPV, JE, MenACYW, MenB, MenC, pneumococcal, polio, rabies, typhoid, varicella, zoster
2024	NSW	Monkeypox, pneumococcal, rabies, RSV
	ACT, TAS, QLD, VIC	RSV
	WA	Hep B, pneumococcal, polio, RSV, varicella, zoster
2025	ACT	JE, monkeypox, pneumococcal, rabies
	NT	Rotavirus, RSV
	QLD	Monkeypox, MenC, rabies
2026	NSW, TAS	Cholera
	WA	MenB, MenC

**Table 3:** Chronological changes in minimum age for pharmacist-administered vaccines (Australia).

Year	State/Territory	Age Change	Vaccine(s)
2018	VIC	↓ ≥16 years	dTpa, influenza
2019	NSW, QLD	↓ ≥16 years	Influenza
	QLD	↓ ≥16 years	dTpa, MMR, influenza
	TAS	↓ ≥10 years	Influenza
2020	ACT, SA, VIC, QLD, NT	↓ ≥10 years	Influenza
	VIC	↓ ≥15 years	dTpa, MMR
2022	WA	↓ ≥5 years, ↓ ≥11 years	Influenza, dTpa
	SA	↓ ≥5 years	Influenza
	NSW	↓ ≥12 years, ↓ ≥5 years	dTpa, MMR, influenza
2023	ACT	↓ ≥5 years	dTpa, MMR, influenza, MenACYW
	NT	↓ ≥5 years	COVID-19, dTpa, MMR, influenza
	QLD	↓ ≥5 years	All PAVs (except COVID-19, influenza)
	SA	↓ ≥5 years	Multiple vaccines
	Tas	↓ ≥10 years	COVID-19, dTpa, MMR
	NSW	↓ ≥9 years	HPV
2024	WA, VIC	↓ ≥5 years	Multiple vaccines
2025	ACT	↓ ≥18 years	Zoster
	QLD	↓ ≥2 years	RSV
	SA	Without age restriction	Multiple vaccines
2026	ACT, NSW, TAS, WA	↓ ≥2 years	Influenza
	QLD	Without age restriction	Multiple vaccines

Note: ↓ = decreased.

## Data Availability

The authors affirm that they had complete access to all data used in this study. The data for this review consisted of publicly available legislative and regulatory documents sourced from official Australian government websites across all states and territories.

## Supplementary Materials

The following supporting information can be downloaded at: https://www.mdpi.com/article/10.3390/pharmacy14040092/s1, Table S1: Pharmacist vaccination formulary in Australia (December 2014 to April 2026).
