# Supplementary material for: The Evolution of Pharmacist Administered Vaccinations in Australia: A Narrative Review of Legislation and Regulatory Documents
Source: Pharmacy (Basel). 2026 Jun 26;14(4):92. doi: 10.3390/pharmacy14040092 (PMC13415602; doi:10.3390/pharmacy14040092)
Supplement: Supplementary file 1 [file pharmacy-14-00092-s001.zip › pharmacy-4346988-Supplementary Material (updated version).pdf]

Supplementary

Supplementary Table S1: Pharmacist vaccination formulary in Australia (December 2014 to April 2026)

State/ Territory	Month/year	Cholera	COVID-19	dTpa	dTpa-IPV	Hib	Hep A	Hep B	HPV	Influenza	JE	Monkeypox	MMR	MenACYW	MenB	MenC	Pneumococcal	Polio	Rabies	Rota virus	RSV	Typhoid	Varicella	Zoster
ACT	March 2016 [12]	X	X	X	X	X	X	X	X	≥ 18	X	X	X	X	X	X	X	X	X	X	X	X	X	X
	June 2017 [76]	X	X	≥ 18	X	X	X	X	X	≥ 18	X	X	X	X	X	X	X	X	X	X	X	X	X	X
	April 2019 [77]	X	X	≥ 16	X	X	X	X	X	≥ 16	X	X	X	X	X	X	X	X	X	X	X	X	X	X
	May 2020 [46]	X	X	≥ 16	X	X	X	X	X	≥ 10	X	X	≥ 16	X	X	X	X	X	X	X	X	X	X	X
	May 2021 [51]	X	≥ 16	≥ 16	X	X	X	X	X	≥ 10	X	X	≥ 16	X	X	X	X	X	X	X	X	X	X	X
	August 2021[78]	X	≥ 10	≥ 16	X	X	X	X	X	≥ 10	X	X	≥ 16	X	X	X	X	X	X	X	X	X	X	X
	December 2021 [79]	X	≥ 5	≥ 16	X	X	X	X	X	≥ 10	X	X	≥ 16	X	X	X	X	X	X	X	X	X	X	X
	March 2023 [24]	X	≥ 5	≥ 12	X	X	≥ 5	≥ 5	≥ 12	≥ 5	X	X	≥ 12	≥ 14	X	X	X	≥ 5	X	X	X	≥ 5	X	≥ 50
	December 2023 [25]	X	≥ 5	≥ 5	X	≥ 5	≥ 5	≥ 5	≥ 10	≥ 5	X	X	≥ 5	≥ 5	≥ 5	≥ 5	X	≥ 5	X	X	X	≥ 5	≥ 5	≥ 50
	May 2024 [31]	X	≥ 5	≥ 5	X	≥ 5	≥ 5	≥ 5	≥ 10	≥ 5	X	X	≥ 5	≥ 5	≥ 5	≥ 5	X	≥ 5	X	X	≥ 60	≥ 5	≥ 5	≥ 50
	March 2025[80]	X	≥ 5	≥ 5	X	≥ 5	≥ 5	≥ 5	≥ 10	≥ 5	≥ 5	≥ 16	≥ 5	≥ 5	≥ 5	≥ 5	≥ 50/70*	≥ 5	≥ 5	X	≥ 60 and Pre	≥ 5	≥ 5	≥ 18
	March 2026[48]	X	≥ 5	≥ 5	X	≥ 5	≥ 5	≥ 5	≥ 10	≥ 2	≥ 5	≥ 16	≥ 5	≥ 5	≥ 5	≥ 5	≥ 50/70*	≥ 5	≥ 5	X	≥ 60 and Preg	≥ 5	≥ 5	≥ 18

	Month/year	Cholera	COVID-19	dTpa	dTpa-IPV	Hib	Hep A	Hep B	HPV	Influenza	JE	Monkey	MMR	MenACYW	MenB	MenC	Pneumococcal	Polio	Rabies	Rota virus	RSV	Typhoid	Varicella	Zoster
NSW	March 2015 [8]	X	X	X	X	X	X	X	X	≥ 18	X	X	X	X	X	X	X	X	X	X	X	X	X	X
	January 2019 [39]	X	X	≥ 16	X	X	X	X	X	≥ 16	X	X	≥ 16	X	X	X	X	X	X	X	X	X	X	X
	March 2020 [81]	X	X	≥ 16	X	X	X	X	X	≥ 10	X	X	≥ 16	X	X	X	X	X	X	X	X	X	X	X
	July 2021 [82]	X	≥ 18	≥ 16	X	X	X	X	X	≥ 10	X	X	≥ 16	X	X	X	X	X	X	X	X	X	X	X
	November 2022 [41]	X	≥ 5	≥ 12	X	X	≥ 5	≥ 5	≥ 12	≥ 5	≥ 5	X	≥ 12	≥ 14	X	X	X	≥ 5	X	X	X	≥ 5	X	≥ 50
	September 2023 [38]	X	≥ 5	≥ 5	X	≥ 5	≥ 5	≥ 5	≥ 9	≥ 5	≥ 5	X	≥ 5	≥ 5	≥ 5	X	X	≥ 5	X	X	X	≥ 5	≥ 5	≥ 50
	October 2023 [38]	X	≥ 5	≥ 5	X	≥ 5	≥ 5	≥ 5	≥ 9	≥ 5	≥ 5	X	≥ 5	≥ 5	≥ 5	≥ 5	X	≥ 5	X	X	X	≥ 5	≥ 5	≥ 18
	December 2024 [29]	X	≥ 5	≥ 5	X	≥ 5	≥ 5	≥ 5	≥ 9	≥ 5	≥ 5	≥ 16	≥ 5	≥ 5	≥ 5	≥ 5	≥ 50/70*	≥ 5	≥ 5	X	≥ 60	≥ 5	≥ 5	≥ 18
	April 2026 [43]	≥ 5	≥ 5		X	≥ 5	≥ 5	≥ 5	≥ 9	≥ 2	≥ 5	≥ 16	≥ 5	≥ 5	≥ 5	≥ 5	≥ 50/70*	≥ 5	≥ 5	X	≥ 60XX	≥ 5	≥ 5	≥ 18
	Month/year	Cholera	COVID-19	dTpa	dTpa-IPV	Hib	Hep A	Hep B	HPV	Influenza	JE	Monkey	MMR	MenACYW	MenB	MenC	Pneumococcal	Polio	Rabies	Rota virus	RSV	Typhoid	Varicella	Zoster
NT	April 2015 [10]	X	X	≥ 16	X	X	X	X	X	≥ 16	X	X	≥ 16	X	X	X	X	X	X	X	X	X	X	X
	May 2020 [47]	X	X	≥ 16	X	X	X	X	X	≥ 10	X	X	≥ 16	X	X	X	X	X	X	X	X	X	X	X
	February 2021 [53]	X	≥ 16	≥ 16	X	X	X	X	X	≥ 10	X	X	≥ 16	X	X	X	X	X	X	X	X	X	X	X

	Month/year	Cholera	COVID-19	dTpa	dTpa-IPV	Hib	Hep A	Hep B	HPV	Influenza	JE	Monkey	MMR	MenACYW	MenB	MenC	Pneumococcal	Polio	Rabies	Rota virus	RSV	Typhoid	Varicella	Zoster
	December 2023[26]	X	≥ 5	≥ 5	X	≥ 5	≥ 5	≥ 5	≥ 5	≥ 5	≥ 2/9 months	≥ 18	≥ 5	≥ 5	≥ 5	X	≥ 5	≥ 5	X	X	X	X	≥ 5	≥ 5
	January 2025 [37]		≥ 5	≥ 5	X	≥ 5	≥ 5	≥ 5	≥ 5	≥ 5	≥ 5	≥ 18	≥ 5	≥ 5	≥ 5	X	≥ 5	≥ 5	X	≥ 5	≥ 5	X	≥ 5	≥ 5
	Month/year	Cholera	COVID-19	dTpa	dTpa-IPV	Hib	Hep A	Hep B	HPV	Influenza	JE	Monkey	MMR	MenACYW	MenB	MenC	Pneumococcal	Polio	Rabies	Rota virus	RSV	Typhoid	Varicella	Zoster
QLD	March 2016 [14]	X	X	≥ 18	X	X	X	X	X	≥ 18	X	X	≥ 18	X	X	X	X	X	X	X	X	X	X	X
	April 2019 [45]	X	X	≥ 16	≥ 16	X	X	X	X	≥16	X	X	≥ 16	X	X	X	X	X	X	X	X	X	X	X
	February 2020 [19]	≥ 16	X	≥ 16	≥ 16	≥ 16	≥ 16	X	X	≥ 10	X	X	≥ 16	≥ 16	X	X	X	≥ 16	X	X	X	X	X	X
	August 2021 [40]	≥ 16	≥ 16	≥ 16	≥ 16	≥ 16	≥ 16	X	X	≥ 10	X	X	≥ 16	≥ 10	X	X	≥ 16	≥ 16	X	X	X	X	X	X
	September 2023 [27]	≥ 2	> 5	≥ 2	≥ 2	≥ 2	≥ 2	≥ 2	≥ 2	≥ 6 months	≥ 2	X	≥ 2	≥ 2	≥ 2	X	≥ 2	≥ 2	X	X	X	≥ 2	≥ 2	≥ 2
	May 2024 [33]	≥ 2	> 5	≥ 2	≥ 2	≥ 2	≥ 2	≥ 2	≥ 2	≥ 6 months	≥ 2	X	≥ 2	≥ 2	≥ 2	X	≥ 2	≥ 2	X	X	≥ 60	≥ 2	≥ 2	≥ 2
	May 2025[83]	≥ 2	≥ 5	≥ 2	≥ 2	≥ 2	≥ 2	≥ 2	≥ 2	≥ 6 months	≥ 2	≥ 2	≥ 2	≥ 2	≥ 2	≥ 2	≥ 2	≥ 2	≥ 2	X	≥ 2	≥ 2	≥ 2	≥ 2
	March 2026[84]	Pharmacist authorised to administer any schedule 4 immunisation medicine within their individual scope of practice and without age restrictions.																						
	Month/year	Cholera	COVID-19	dTpa	dTpa-IPV	Hib	Hep A	Hep B	HPV	Influenza	JE	Monkey	MMR	MenACYW	MenB	MenC	Pneumococcal	Polio	Rabies	Rota virus	RSV	Typhoid	Varicella	Zoster
SA	February 2015 [9]	X	X	X	X	X	X	X	X	≥ 16	X	X	X	X	X	X	X	X	X	X	X	X	X	X

	March 2017 [9]	X	X	X	≥ 16	X	X	X	X	≥ 16	X	X	≥ 16	X	X	X	X	X	X	X	X	X	X	X
	February 2018 [9]	X	X	X	≥ 16	X	X	X	X	≥ 16	X	X	≥ 16	≥ 16	X	X	X	X	X	X	X	X	X	X
	October 2018 [9]	X	X	X	≥ 16	X	X	X	X	≥ 16	X	X	≥ 16	≥ 16	≥ 16	≥ 16	X	X	X	X	X	X	X	X
	March 2020 [9]	X	X	X	≥ 16	X	X	X	X	≥ 10	X	X	≥ 16	≥ 16	≥ 16	≥ 16	X	X	X	X	X	X	X	X
	February 2021 [9]	X	≥ 16	X	≥ 16	X	X	X	X	≥ 10	X	X	≥ 16	≥ 16	≥ 16	≥ 16	X	X	X	X	X	X	X	X
	April 2022 [9]	X	≥ 16	X	≥ 16	X	X	X	X	≥ 5	X	X	≥ 16	≥ 16	≥ 16	≥ 16	X	X	X	X	X	X	X	X
	April 2023 [9]	X	≥ 16	≥ 10	≥ 10	≥ 10	≥ 10	≥ 10	≥ 10	≥ 5	≥ 5		≥ 10	≥ 10	≥ 10	≥ 10	X	≥ 10	X	X	X	X	≥ 10	≥ 50
	December 2023 [9]	X	≥ 5	≥ 5	≥ 5	≥ 10	≥ 10	≥ 5	≥ 5	≥ 5	≥ 5	X	≥ 5	≥ 5	≥ 5	≥ 5	≥ 5	≥ 5	X	X	X	X	≥ 5	≥ 50
	January 2025 [36]	Pharmacists authorised to administer any vaccine within their individual scope of practice																						
	Month/year	Cholera	COVID-19	dTpa	dTpa-IPV	Hib	Hep A	Hep B	HPV	Influenza	JE	Monkey	MMR	MenACYW	MenB	MenC	Pneumococcal	Polio	Rabies	Rota virus	RSV	Typhoid	Varicella	Zoster
TAS	February 2016 [13]	X	X	X	X	X	X	X	X	≥ 18	X	X	X	X	X	X	X	X	X	X	X	X	X	X
	September 2019 [85]	X	X	≥ 16	X	X	X	X	X	≥ 10	X	X	≥ 16	X	X	X	X	X	X	X	X	X	X	X
	August 2021 [85]	X	≥ 16	≥ 16	X	X	X	X	X	≥ 10	X	X	≥ 16	X	X	X	X	X	X	X	X	X	X	X
	March 2023 [28]	X	5 to 10**	5 to 10** or	X	5 to 10** or	5 to 10** or	5 to 10** or	5 to 10** or	5 to 10**	5 to 10** or ≥	X	5 to 10** or	5 to 10** or	5 to 10** or	5 to 10** or	5 to 10** or ≥	5 to 10** or	5 to 10** or	X	X	5 to 10** or	5 to 10** or	≥ 10

			or ≥ 10	≥ 10*** or ≥ 16		≥ 10***	≥ 10***	≥ 10***	≥ 10***	or ≥ 10	10***		≥ 10*** or ≥ 16	≥ 10***	≥ 10***	≥ 10***	10***	≥ 10***	≥ 10***			≥ 10***	≥ 10***	
	January 2024 [13]	X	5 to 10*** or ≥ 10	≥ 10	X	≥ 10	≥ 10	≥ 10	≥ 10	5 to 10** or ≥ 10	≥ 10***	X	≥ 10	≥ 10	≥ 10	≥ 10	≥ 10	≥ 10	≥ 10***	X	≥ 60/18	≥ 10***	≥ 10	≥ 18
	March 2026[44]	≥ 5***	≥ 5	≥ 5	X	≥ 5	≥ 5	≥ 5	≥ 9	≥ 2	≥ 5***	X	≥ 5	≥ 5	≥ 5	≥ 5	≥ 5	≥ 5	≥ 5***	X	≥ 60/50/18	≥ 5***	≥ 5	≥ 18
	Month/year	Cholera	COVID-19	dTpa	dTpa-IPV	Hib	Hep A	Hep B	HPV	Influenza	JE	Monkey	MMR	MenACYW	MenB	MenC	Pneumococcal	Polio	Rabies	Rota virus	RSV	Typhoid	Varicella	Zoster
VIC	May 2016 [15]	X	X	≥ 18	X	X	X	X	X	≥ 18	X	X	X	X	X	X	X	X	X	X	X	X	X	X
	October 2018 [16]	X	X	≥ 16	X	X	X	X	X	≥ 16	X	X	≥ 16	X	X	X	X	X	X	X	X	X	X	X
	April 2020 [20]	X	X	≥ 15	X	X	X	X	X	≥ 10	X	X	≥ 15	≥ 15	X	X	X	X	X	X	X	X	X	X
	May 2021 [54]	X	****	≥ 15	X	X	X	X	X	≥ 10	X	X	≥ 15	≥ 15	X	X	X	X	X	X	X	X	X	X
	October 2022 [42]	X	****	≥ 12	X	X	X	X	≥ 12	≥ 5	≥ 5	≥ 5	≥ 15	≥ 15	X	X	≥ 50	X	X	X	X	X	X	≥ 50
	October 2024 [32]	X	≥ 5	≥ 5	X	X	X	X	≥ 5	≥ 5	≥ 5	≥ 5	≥ 5	≥ 5	X	X	≥ 5	X	X	X	≥ 5	X	X	≥ 5
	Month/year	Cholera	COVID-19	dTpa	dTpa-IPV	Hib	Hep A	Hep B	HPV	Influenza	JE	Monkey	MMR	MenACYW	MenB	MenC	Pneumococcal	Polio	Rabies	Rota virus	RSV	Typhoid	Varicella	Zoster
WA	December 2014 [86]	X	X	X	X	X	X	X	X	≥ 18	X	X	X	X	X	X	X	X	X	X	X	X	X	X

	October 2019 [18]	X	X	≥ 16	X	X	X	X	X	≥18	X	X	≥ 16	≥ 16	X	X	X	X	X	X	X	X	X	X
	November 2021[55]	X	≥ 18	≥ 16	X	X	X	X	X	≥18	X	X	≥ 16	≥ 16	X	X	X	X	X	X	X	X	X	X
	April 2022 [21]	X	≥ 16	≥ 11	X	X	X	X	≥ 11	≥ 5	X	X	≥ 16	≥ 15	X	X	X	X	X	X	X	X	X	X
	August 2024 [35]	X	≥ 5	≥ 5	X	X	X	≥ 5	≥ 5	≥ 5	X	X	≥ 5	≥ 5	X	X	≥ 5	≥ 5	X	X	≥ 5	X	≥ 5	≥ 5
	May 2026[49]	X	≥ 5	≥ 5	X	X	X	≥ 5	≥ 5	≥ 2	X	X	≥ 5	≥ 5	≥ 5	≥ 5	≥ 5	≥ 5	X	X	≥ 5	X	≥ 5	≥ 5

*The eligibility age for the pneumococcal vaccine is 50 years and over for Aboriginal people, and 70 years and over for non-Aboriginal people.

**Authorised Pharmacist immunisers must have additional paediatric authorisation with the department

***Prescription-initiated: Authorised Pharmacist Immunisers can administer upon receipt of a valid prescription from a medical or nurse practitioner

XPharmacists not allowed to administer the vaccine

**** Different age categories based on TGA

Pre: to pregnant people between 28 to 36 weeks gestation

Preg: to pregnant people over 36 weeks gestation

XX: Arexvy for 60 years and over AND for 50-59 years who are at increased risk for RSV disease; Abrysvo for 60 years and over and pregnant women from 28 weeks' gestation for each pregnancy

The number in each box indicates the minimum age (in year) a pharmacist is legally permitted to administer the vaccine.
